# Draft genome sequence of *Streptococcus infantarius* strain UMPGM isolated from individual withdrawing from methamphetamine

**DOI:** 10.1128/mra.00368-26

**Published:** 2026-06-30

**Authors:** Siti Khadijah Kiraman, Norafisah Mohd Arshad, Hajar Fauzan Ahmad

**Affiliations:** 1 Faculty of Industrial Sciences and Technology, University of Malaysia Pahang Al-Sultan Abdullah 294596 , Gambang, Pahang, Malaysia; Portland State University, Portland, Oregon, USA

**Keywords:** methamphetamine, microbiome, addiction, gut microbiota

## Abstract

We report draft the genome sequence of *Streptococcus infantarius* strain UMPGM (1,791,962 bp, guanine-cytosine [GC] content of 37.70%) isolated from an individual recovering from methamphetamine use disorder. The organism was isolated from a stool sample on de Man, Rogosa, and Sharpe agar under anaerobic condition.

## ANNOUNCEMENT

*Streptococcus infantarius* has been increasingly recognized for its association with gastrointestinal conditions, including bacteremia, colorectal neoplasia, and other intestinal disorders (1, 2). Here, we report the draft genome sequence of *S. infantarius* strain UMPGM isolated from a stool sample collected in February 2026 of a 27-year-old male in Pahang, Malaysia with methamphetamine use disorder (MUD). This genome provides strain-level data for gut-derived *S. infantarius* in the context of MUD.

The stool homogenate was 10-fold serially diluted and spread-plated onto de Man, Rogosa, and Sharpe agar and incubated under anaerobic condition with AnaeroPack (Mitsubishi Gas Chemical Co., Inc., Japan) at 37°C overnight (3). The colonies were small, creamy-white, circular, smooth, convex, and glistening with entire margins. A single colony was restreaked under the same conditions. Next, 250 mg of colonies was suspended into 200 µL sterile phosphate-buffered saline solution prior DNA extraction using NucleoSpin Microbial DNA Mini Kit (Macherey-Nagel, Germany) following manufacturer’s instructions.

Library construction used 100 ng of gDNA quantified with the DeNovix dsDNA High-Sensitivity Assay (DeNovix, Inc., Wilmington, DE). Library was constructed using the Illumina DNA Prep Kit (Illumina, San Diego, CA) and sequenced on the Illumina NovaSeq X platform (2 × 150 bp paired-end), yielding 1 Gb of data per sample. Raw reads were processed using fastp v0.23.2 (4), retaining reads ≥50 bp. Genome assembly was performed using the Shovill v1.1.0 pipeline (5), incorporating genome size estimation with KMC v3.2.1 (6), reads subsampling to 150× with Seqtk v1.4 (7), error correction using Lighter v1.1.3 (8), and paired-end merging via FLASH v1.2.11 (9). The pre-processed reads were assembled *de novo* with Unicycler v0.5.1 (10), retaining contigs ≥500 bp. Plasmid and phage sequences were identified using geNomad v1.11.2 (11). Taxonomic was assigned using GTDB-Tk v2.6.1 (12) with GTDB release R226 (13), employing skani v0.3.0 (14). Multilocus sequence typing was performed using mlst v2.32.2 (15) based on the PubMLST scheme. Assembly quality and completeness were assessed using SeqKit v2.3.0 (16) and BUSCO v6.0.0 (17) with the lactobacillales_odb10 data set. Protein-coding genes were predicted using Prodigal v2.6.3 (18) and functionally annotated with eggNOG-mapper v2.1.13 (19). rRNA and tRNA gene counts were obtained from the National Center for Biotechnology Information (NCBI) Prokaryotic Genome Annotation Pipeline (PGAP) annotation (20). Antimicrobial resistance genes and virulence factors were screened using ABRicate v1.2.0 (21) against the CARD, NCBI, ResFinder, VFDB, and Victors (22) databases dated 7 April 2026. Default parameters were used unless specified.

Genome analysis identified the isolate as *Streptococcus infantarius* with 98.43% average nucleotide identity (ANI) to reference genome GCF_000154985.1. mlst v2.23.2 (15) assigned it to the SBSEC scheme where five loci showed exact allele matches, while five showed uncertain matches; therefore, no ST was assigned (Table 1). eggNOG-mapper v2.1.13 (19) revealed multiple genes related to carbohydrate transport systems, including phosphotransferase system components for diverse sugars. Annotated genes related to stress response, such as molecular chaperones (dnaK, dnaJ, groEL), ATP-dependent proteases (Clp family), and oxidative stress enzymes, were present, indicating stress-response features. Virulence screening using ABRicate v1.2.0 (21) identified homologs in the Victors database (22), while no virulence or antimicrobial resistance genes were detected in VFDB, CARD, or ResFinder.

**TABLE 1:** Genome characteristics of *Streptococcus infantarius* strain UMPGM

Parameter	Result		
Total number of reads	12,914,788		
Genome size (bp)	1,791,962		
GC content (%)	37.70%		
Genome coverage	1,081×		
Number of contigs	90		
N50	36,117		
CDS	1,805		
rRNA	3		
tRNA	27		
BUSCO data set	Complete BUSCOs (C)		100.0%
	Single-copy BUSCOs (S)		99.8%
	Duplicated BUSCOs (D)		0.2%
	Fragmented BUSCOs (F)		0.0%
	Missing BUSCOs (M)		0.0%
	Total BUSCO groups searched (n)		402
geNomad v1.11.2 (11)	Two putative plasmid-associated contigs Two prophage regions		

	Associated victors entry	Entry	Coverage/identity
Victors homologs	*Streptococcus pneumoniae* D39	`gi	116515308:668966–669556`
	*Streptococcus pneumoniae* TIGR4	`gi	194172857:96869–97855`
	*Streptococcus pneumoniae* TIGR4	`gi	194172857:224751–227252`
	*Streptococcus pneumoniae* CGSP14	`gi	182682970:476082–477428`
	*Streptococcus pyogenes* M1 GAS	`gi	15674250:1567023–1567904`
	*Streptococcus pneumoniae* D39	`gi	116515308:59174–60472`
	*Streptococcus pneumoniae* TIGR4	`gi	194172857:53070–54617`
	*Streptococcus agalactiae* A909	`gi	76786714:988511–990343`
	*Streptococcus agalactiae* A909	`gi	76786714:1031903–1033465`
	*Streptococcus agalactiae* A909	`gi	76786714:114765–115340`

## Data Availability

This Whole Genome Shotgun project has been deposited at DDBJ/ENA/GenBank under accession number JBVYON000000000. The version described in this paper is version JBVYON010000000. The sequencing data are available in the SRA under accession number SRR37497973, associated with BioProject PRJNA1433185 and BioSample SAMN56357951.

## References

[1] Ouranos K, Gardikioti A, Bakaloudi DR, Mylona EK, Shehadeh F, Mylonakis E. 2023. Association of the *Streptococcus bovis/Streptococcus equinus* complex with colorectal neoplasia: a systematic review and meta-analysis. Open Forum Infect Dis 10:ofad547. doi:10.1093/ofid/ofad547 38023558 PMC10655943

[2] Corredoira J, Coira A, Iñiguez I, Pita J, Varela J, Alonso MP. 2013. Advanced intestinal cancer associated with *Streptococcus infantarius* (former *S. bovis* II/1) sepsis. Int J Clin Pract 67:1358–1359. doi:10.1111/ijcp.12190 24246215

[3] Jans C, Gerber A, Bugnard J, Njage PMK, Lacroix C, Meile L. 2012. Novel *Streptococcus infantarius* subsp. infantarius variants harboring lactose metabolism genes homologous to *Streptococcus thermophilus*. Food Microbiol 31:33–42. doi:10.1016/j.fm.2012.02.001 22475940

[4] Chen S, Zhou Y, Chen Y, Gu J. 2018. Fastp: an ultra-fast all-in-one FASTQ preprocessor. Bioinformatics 34:i884–i890. doi:10.1093/bioinformatics/bty560 30423086 PMC6129281

[5] Seemann T. 2017. Shovill: faster SPAdes assembly of Illumina isolates. 1.1.0. GitHub

[6] Kokot M, Dlugosz M, Deorowicz S. 2017. KMC 3: counting and manipulating k-mer statistics. Bioinformatics 33:2759–2761. doi:10.1093/bioinformatics/btx304 28472236

[7] H L. 2012. Seqtk: toolkit for processing sequences in FASTA/Q formats. 1.4. GitHub

[8] Song L, Florea L, Langmead B. 2014. Lighter: fast and memory-efficient sequencing error correction without counting. Genome Biol 15:509. doi:10.1186/s13059-014-0509-9 25398208 PMC4248469

[9] Magoč T, Salzberg SL. 2011. FLASH: fast length adjustment of short reads to improve genome assemblies. Bioinformatics 27:2957–2963. doi:10.1093/bioinformatics/btr507 21903629 PMC3198573

[10] Wick RR, Judd LM, Gorrie CL, Holt KE. 2017. Unicycler: resolving bacterial genome assemblies from short and long sequencing reads. PLoS Comput Biol 13:e1005595. doi:10.1371/journal.pcbi.1005595 28594827 PMC5481147

[11] Camargo AP, Roux S, Schulz F, Babinski M, Xu Y, Hu B, Chain PSG, Nayfach S, Kyrpides NC. 2023. Identification of mobile genetic elements with geNomad. Nat Biotechnol 42:1303–1312. doi:10.1038/s41587-023-01953-y 37735266 PMC11324519

[12] Chaumeil PA, Mussig AJ, Hugenholtz P, Parks DH. 2022. GTDB-Tk v2: memory friendly classification with the genome taxonomy database. Bioinformatics 38:5315–5316. doi:10.1093/bioinformatics/btac672 36218463 PMC9710552

[13] Parks DH, Chuvochina M, Rinke C, Mussig AJ, Chaumeil PA, Hugenholtz P. 2022. GTDB: an ongoing census of bacterial and archaeal diversity through a phylogenetically consistent, rank normalized and complete genome-based taxonomy. Nucleic Acids Res 50:D785–D794. doi:10.1093/nar/gkab776 34520557 PMC8728215

[14] Shaw J, Yu YW. 2023. Fast and robust metagenomic sequence comparison through sparse chaining with skani. Nat Methods 20:1661–1665. doi:10.1038/s41592-023-02018-3 37735570 PMC10630134

[15] Seemann T. 2023. mlst. 2.32.2. GitHub

[16] Shen W, Le S, Li Y, Hu F. 2016. SeqKit: a cross-platform and ultrafast toolkit for FASTA/Q file manipulation. PLoS One 11:e0163962. doi:10.1371/journal.pone.0163962 27706213 PMC5051824

[17] Manni M, Berkeley MR, Seppey M, Zdobnov EM. 2021. BUSCO: assessing genomic data quality and beyond. Curr Protoc 1:e323. doi:10.1002/cpz1.323 34936221

[18] Hyatt D, Chen G-L, Locascio PF, Land ML, Larimer FW, Hauser LJ. 2010. Prodigal: prokaryotic gene recognition and translation initiation site identification. BMC Bioinformatics 11:119. doi:10.1186/1471-2105-11-119 20211023 PMC2848648

[19] Cantalapiedra CP, Hernández-Plaza A, Letunic I, Bork P, Huerta-Cepas J. 2021. eggNOG-mapper v2: functional annotation, orthology assignments, and domain prediction at the metagenomic scale. Mol Biol Evol 38:5825–5829. doi:10.1093/molbev/msab293 34597405 PMC8662613

[20] Tatusova T, DiCuccio M, Badretdin A, Chetvernin V, Nawrocki EP, Zaslavsky L, Lomsadze A, Pruitt KD, Borodovsky M, Ostell J. 2016. NCBI prokaryotic genome annotation pipeline. Nucleic Acids Res 44:6614–6624. doi:10.1093/nar/gkw569 27342282 PMC5001611

[21] Seemann T. 2025. ABRicate: mass screening of contigs for antimicrobial resistance or virulence genes. 1.2.0. GitHub

[22] Sayers S, Li L, Ong E, Deng S, Fu G, Lin Y, Yang B, Zhang S, Fa Z, Zhao B, et al.. 2019. Victors: a web-based knowledge base of virulence factors in human and animal pathogens. Nucleic Acids Res 47:D693–D700. doi:10.1093/nar/gky999 30365026 PMC6324020

